# EXPLORING CAREGIVING PATHS AMONG RACIALLY DIVERSE FAMILY CAREGIVERS OF OLDER ADULTS WITH DEMENTIA

**DOI:** 10.1093/geroni/igae098.3460

**Published:** 2024-12-31

**Authors:** Jung-Ah Lee, Hyun Jung Kim, Young-Shin Lee

**Affiliations:** University of California Irvine, Irvine, California, United States; Seattle University, Seattle, California, United States; San Diego State University, San Diego, California, United States

## Abstract

Ethnic minority family caregivers who directly care for community-dwelling older adults experiencing dementia, navigate distinct paths in their caregiving journey. Our aim was to discern the unique aspects of caregiving experienced by ethnic minorities in comparison to White caregivers. The study employed a descriptive qualitative design utilizing thematic analysis. Participants were recruited from diverse California ethnic communities. Bilingual nursing students offered emotional support to home-bound caregivers through weekly calls for two months during the pandemic crisis. Twenty-three participants included 7 White, 8 Latino, 5 Korean, and 3 Vietnamese caregivers. The mean age was 60, ranging from 27 to 79 years old. Eighty-one percent of minority persons with dementia (PWD) were Medicaid beneficiaries, while none of the White PWD were low-income. Significant differences in caregiving experiences were identified across three key areas: problem-solving skills, social boundaries, and dynamics between PWD and their caregivers. White caregivers reported more active problem-solving skills, establishing social boundaries that extended beyond their immediate family members, and dedicating more time to engaging PWD in shared activities. Conversely, minority caregivers with limited English proficiency exhibited a tendency to endure problems, carefully constructed social boundaries with selective membership, and seemed to prefer solitary activities (e.g., watching TV) over actively engaging with PWD (e.g., gardening). The findings showed that low-income minority caregivers had lower problem-solving skills, limited social boundaries, and less PWD engagement at home compared to counterparts. We underscore the importance of culturally sensitive support and tailored interventions in addressing the diverse needs of caregivers across different ethnic backgrounds.

